# Supplementary material for: Soil Fungal Diversity and Ecology Assessed Using DNA Metabarcoding along a Deglaciated Chronosequence at Clearwater Mesa, James Ross Island, Antarctic Peninsula
Source: Biology (Basel). 2023 Feb 9;12(2):275. doi: 10.3390/biology12020275 (PMC9953209; doi:10.3390/biology12020275)
Supplement: Supplementary file 1 [file biology-12-00275-s001.zip › biology-1937047-supplementary.pdf]

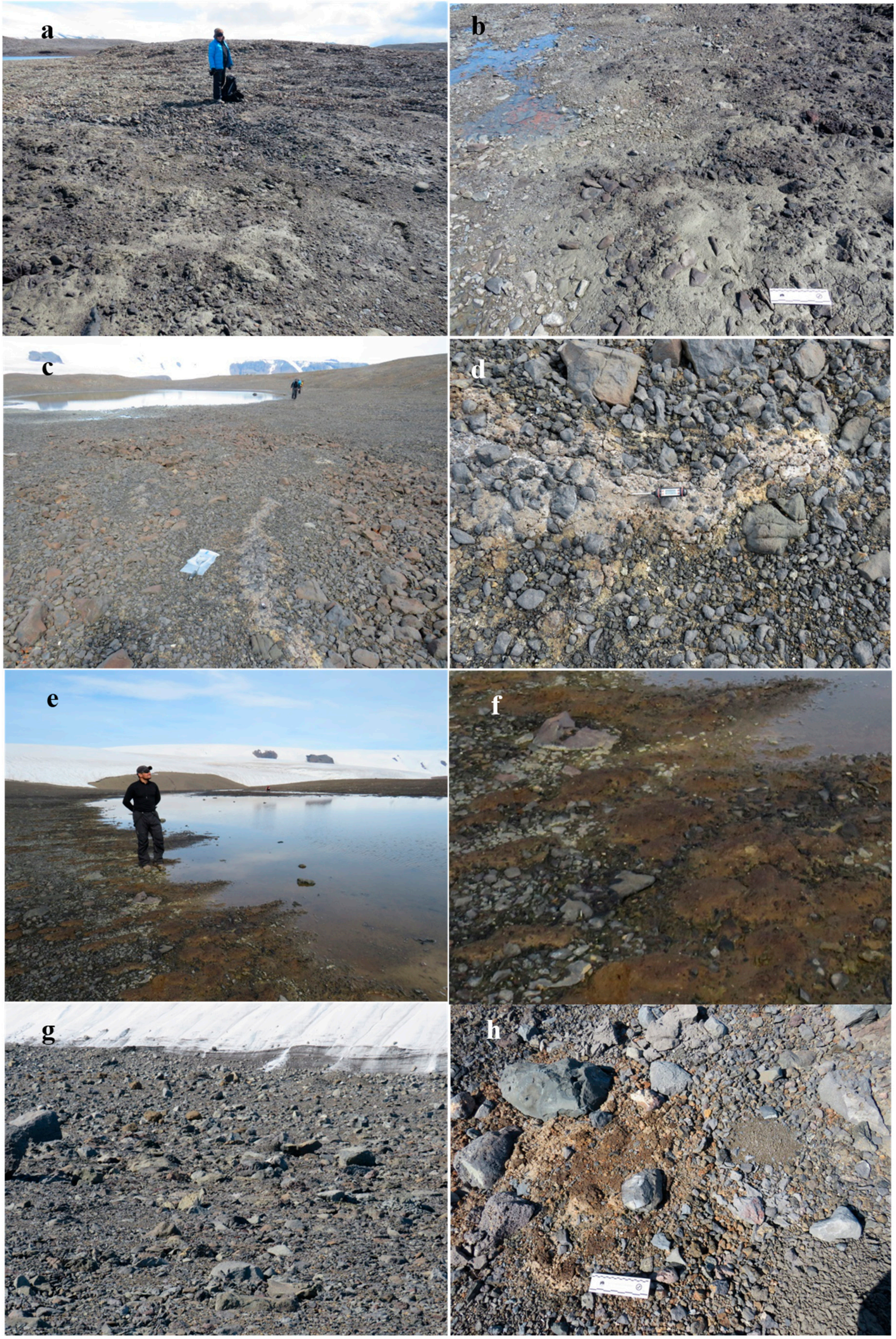

Figure S1. General and detailed images of the soils sampled at (**a, b**) site 1, (**c, d**) site 2, (**e, f**) site 3 and (**g, h**) site 4 on Clearwater Mesa, James Ross Island, Antarctic Peninsula. Photos taken by Juan Lirio.

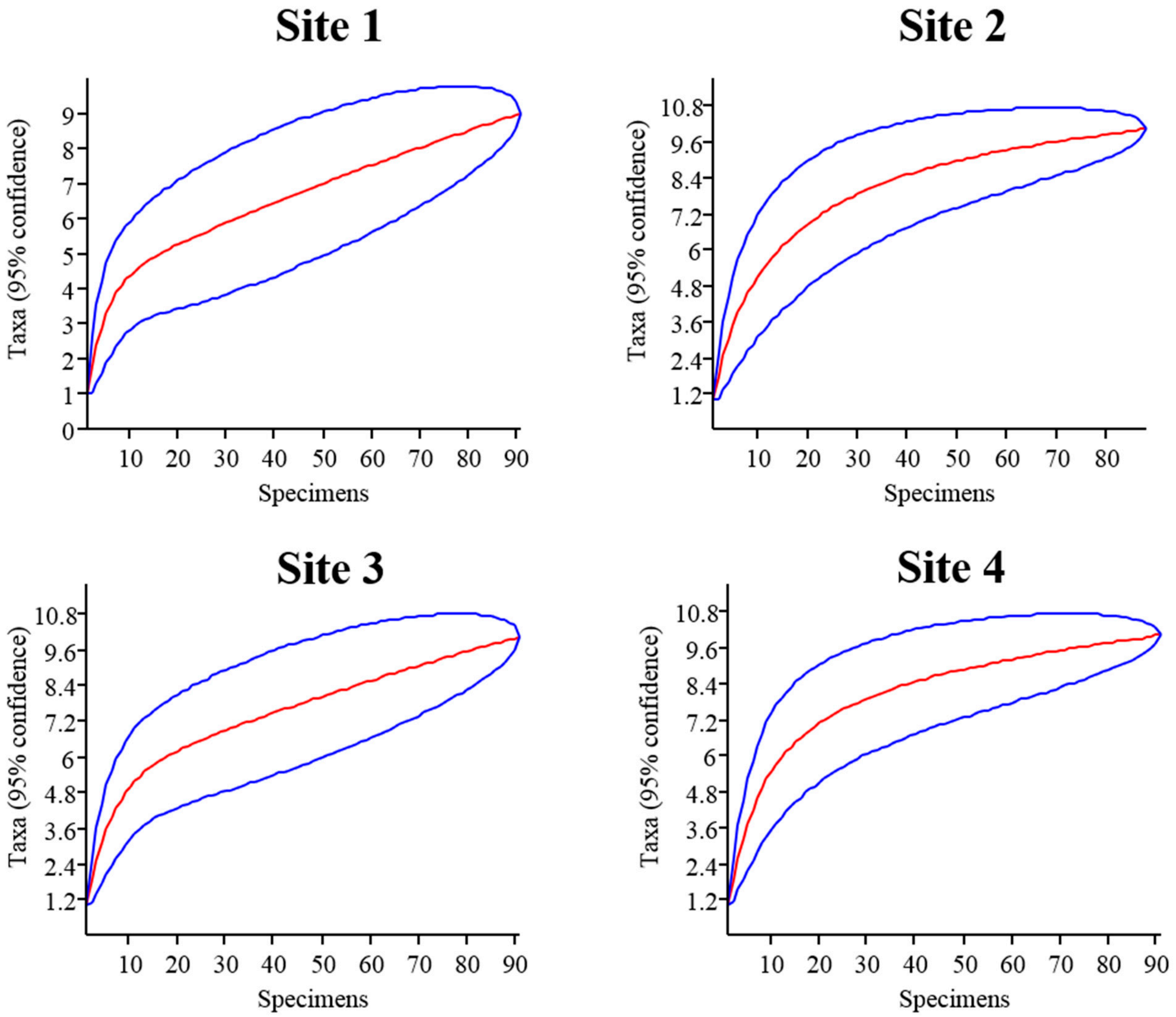

Figure S2. Rarefaction curves, with 95% confidence limits, of fungal amplicon sequence variants (ASVs) obtained from the four soils sampled at Clearwater Mesa, James Ross Island, Antarctic Peninsula.

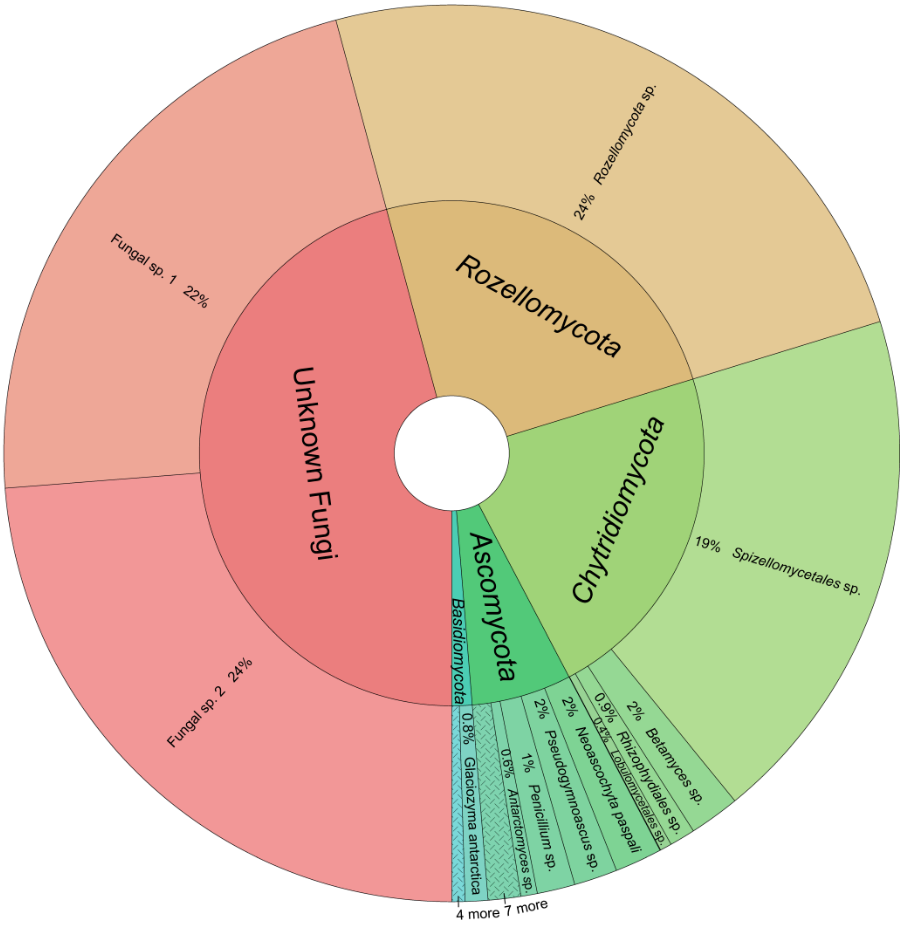
Rozellomycota
24% Rozellomycota sp.
Unknown Fungi
Fungal sp. 1 22%
Fungal sp. 2 24%
Chytridiomycota
19% Spizellomycetales sp.
Ascomycota
Basidiomycota
0.8% Glaciozyma antarctica
0.6% Antarctomyces sp.
1% Penicillium sp.
2% Pseudogymnoascus sp.
2% Neoascochyta paspali
0.9% Rhizophydiales sp.
Lobulomycetales sp.
2% Betamyces sp.
4 more
7 more

(a)

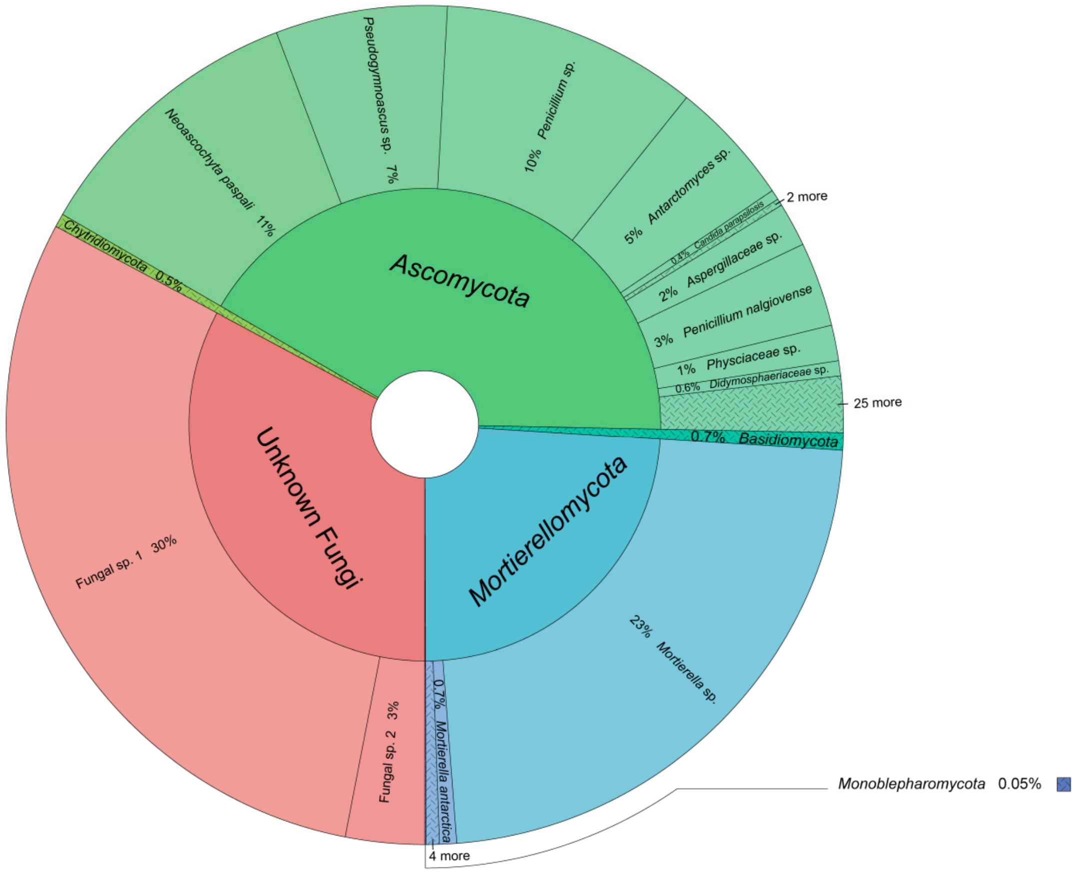
Ascomycota
Neoascochyta paspali 11%
Pseudogymnoascus sp. 7%
10% Penicillium sp.
5% Antarctomyces sp.
2 more
Candida parapsilosis
2% Aspergillaceae sp.
3% Penicillium nalgiovense
1% Physciaceae sp.
0.6% Didymosphaeriaceae sp.
25 more
0.7% Basidiomycota
Mortierellomycota
23% Mortierella sp.
0.7% Mortierella antarctica
4 more
Monoblepharomycota 0.05%
Unknown Fungi
Fungal sp. 1 30%
Fungal sp. 2 3%
Chytridiomycota 0.5%

(**b**)

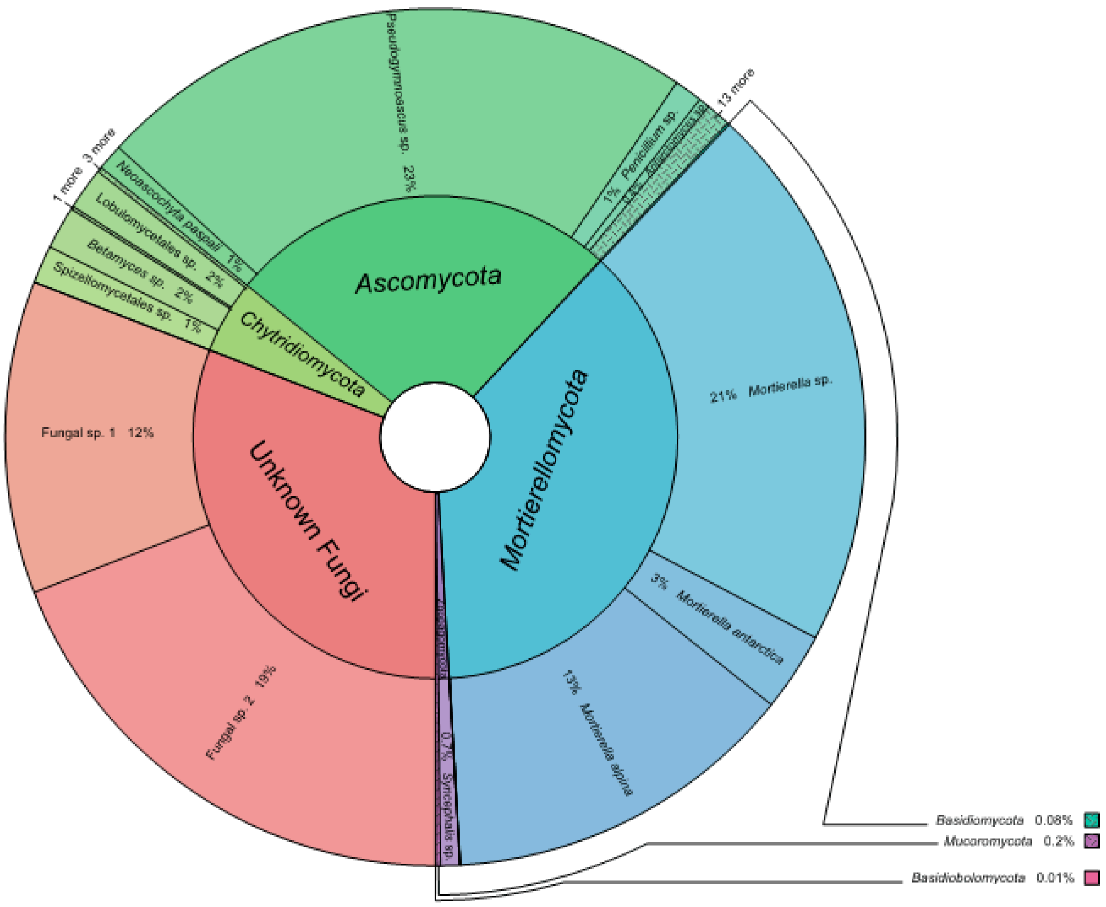
Ascomycota
Pseudogymnoascus sp. 23%
1% Penicillium sp.
13 more
3 more
1 more
Neoascochyta paspali 1%
Lobulomycetales sp. 2%
Betamyces sp. 2%
Spizellomycetales sp. 1%
Chytridiomycota
Mortierellomycota
21% Mortierella sp.
3% Mortierella antarctica
13% Mortierella alpina
Unknown Fungi
Fungal sp. 1 12%
Fungal sp. 2 19%
0.7% Syncephalis sp.
Basidiomycota 0.08%
Mucoromycota 0.2%
Basidiobolomycota 0.01%

(**c**)

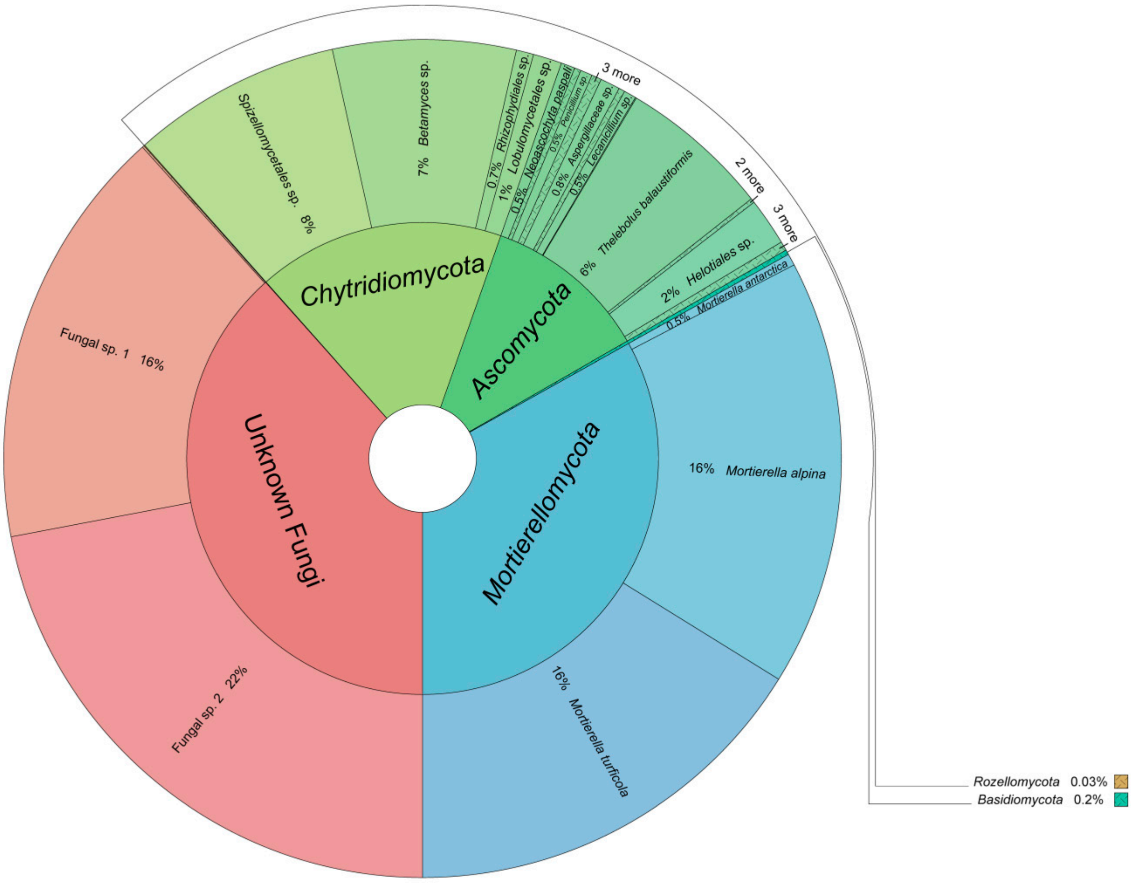

(**d**)

Figure S3. Krona chart showing the abundances of different fungal taxonomic levels detected in soil samples from (**a**) site 1, (**b**) site 2, (**c**) site 3 and (**d**) site 4 at Clearwater Mesa, James Ross Island, Antarctic Peninsula.

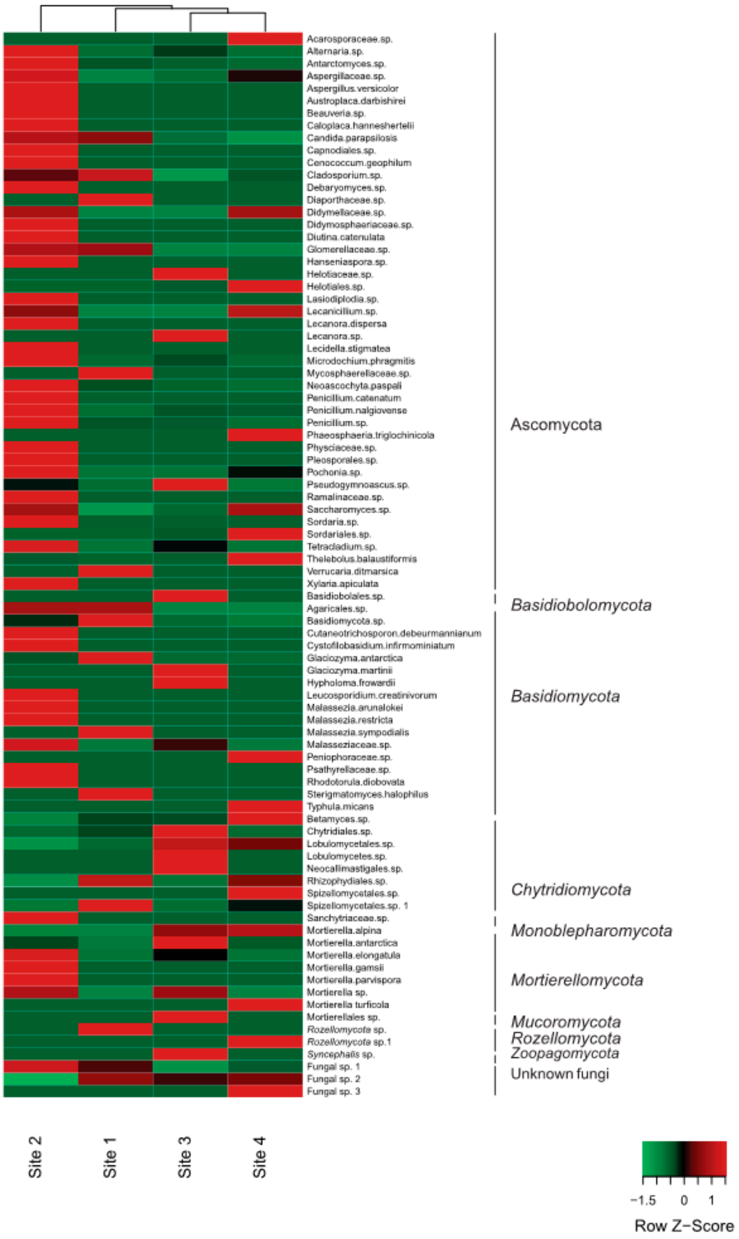

Figure S4. Heat map of fungal assemblage relative abundances detected in the four soils sampled. The color intensities range from red (highest relative abundance) to green (lowest relative abundance). These values represent percentages of DNA fungal reads from sites 1, 2, 3 and 4 at Clearwater Mesa, James Ross Island, Antarctic Peninsula. The heatmap of ASV abundance was created using the following parameters: Average Linkage, Spearman Rank Correlation, and Z-score among samples for each ASV.

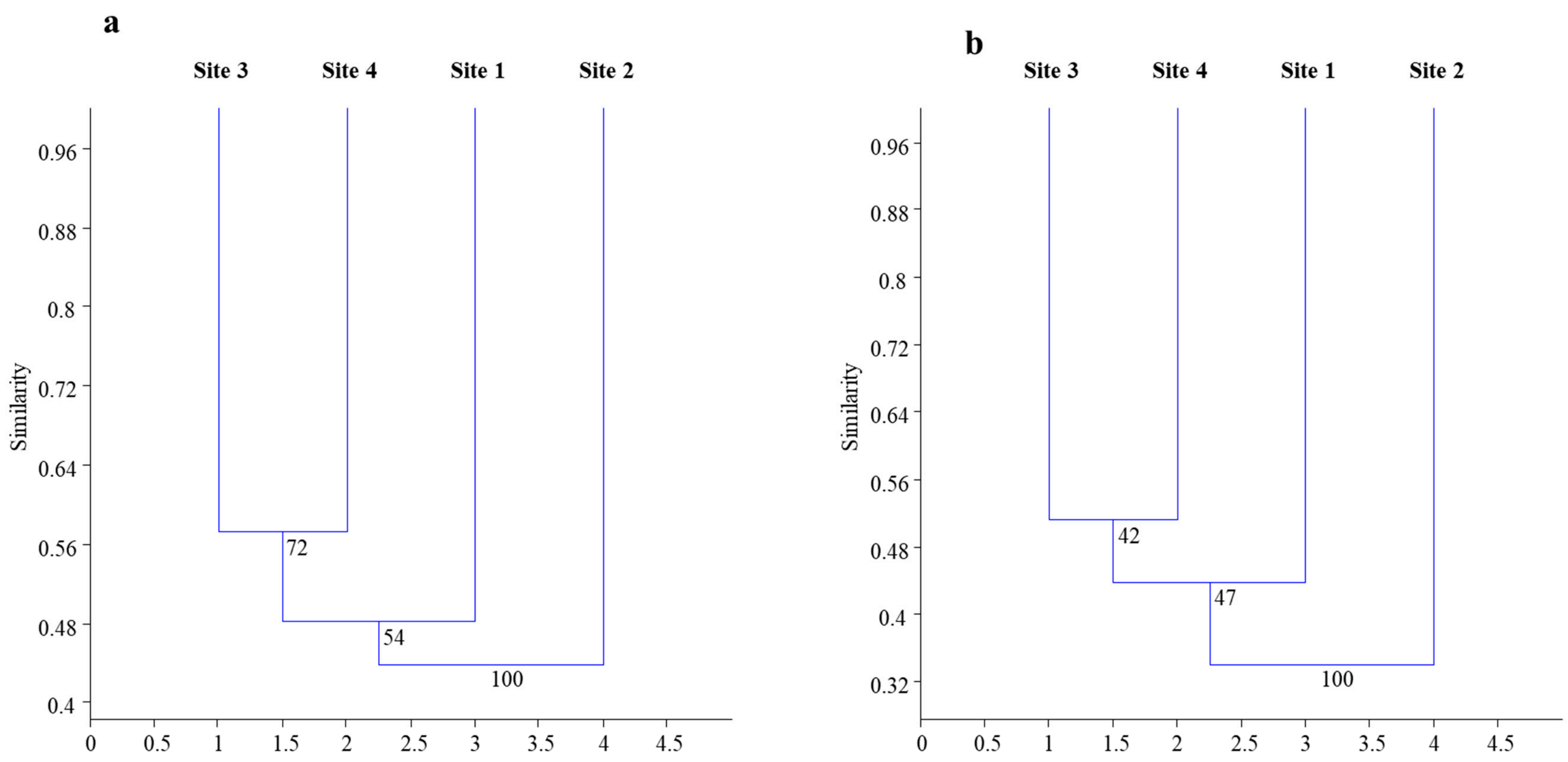

Figure S5. Dendrograms showing the Sorensen (**a**) and (**b**) Bray-Curtis similarity measures for the fungal assemblages detected in soils sampled from the four sites on Clearwater Mesa, James Ross Island, Antarctic Peninsula. The results were obtained with 95% confidence and bootstrap values calculated from 1,000 iterations.

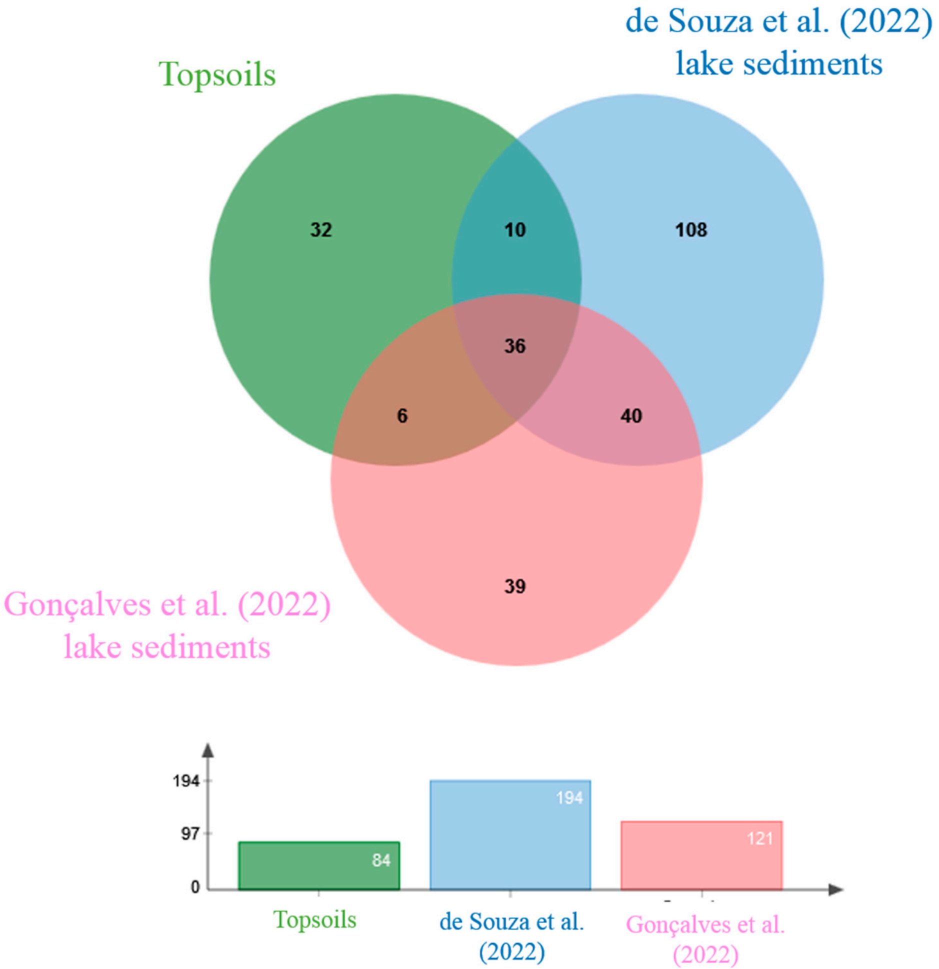

Common fungi in topsoils and lake sediments (de Souza et al. 2022 and Gonçalves et al. 2022):

Pseudogymnoascus sp.
Neoascochyta paspali
Penicillium sp.
Thelebolus balaustiformis
Antarctomyces sp.
Penicillium nalgiovense
Aspergillaceae sp.
Ciliophora sp.
Helotiales sp.
Candida parapsilosis
Didymosphaeriaceae sp.
Cladosporium sp.
Didymellaceae sp.
Microdochium phragmitis
Saccharomyces sp.
Tetracladium sp.
Glaciozyma antarctica
Malassezia restricta
Cutaneotrichosporon debeurmannianum
Malassezia arunalokei
Leucosporidium creatinivorum
Mortierella alpina
Mortierella antarctica
Betamyces sp.
Lobulomycetales sp.
Spizellomycetales sp.
Rhizophydiales sp.
Chytridiales sp.
Lobulomycetes sp.
Basidiobolales sp.
Agaricales sp.
Basidiomycota sp.
Sordariales sp.
Mortierellales sp.
Fungal sp.

Figure S6. Venn diagram showing the overlap in distribution of fungal amplicon sequence variants (ASVs) among the soils and lake sediments analyzed in the current study and by de Souza et al. (2022) and Gonçalves et al. (2022) at Clearwater Mesa, James Ross Island, Antarctic Pensinsula.

Table S1. Relative abundances of the fungal amplicon sequence variants (ASVs) detected in four soils sampled from Clearwater Mesa, James Ross Island, Antarctic Peninsula.

				Relative abundance (%) of fungal ASVs			
**Database**	**Kingdom**	**Phylum**	**Fungal amplicon variant sequence (ASV)**	**Site 1**	**Site 2**	**Site 3**	**Site 4**
UNITE	Fungi	*Ascomycota*	*Pseudogymnoascus* sp.	1.529	6.627	22.650	0.252
			Neoascochyta paspali	1.663	10.873	0.991	0.535
			Penicillium sp.	1.334	9.887	1.059	0.462
			Thelebolus balaustiformis	0.000	0.136	0.064	5.843
			Antarctomyces sp.	0.579	4.828	0.415	0.214
			Penicillium nalgiovense	0.000	3.261	0.310	0.237
			Aspergillaceae sp.	0.074	1.696	0.251	0.764
			Ciliophora sp.	2.334	0.000	0.007	0.000
			Helotiales sp.	0.000	0.000	0.029	1.837
			Physciaceae sp.	0.000	1.375	0.000	0.000
			Candida parapsilosis	0.320	0.387	0.133	0.073
			Lecanicillium sp.	0.000	0.369	0.000	0.471
			Didymosphaeriaceae sp.	0.000	0.579	0.000	0.000
			Cladosporium sp.	0.265	0.174	0.020	0.097
			Diaporthaceae sp.	0.333	0.000	0.000	0.000
			Ramalinaceae sp.	0.000	0.294	0.000	0.000
			Didymellaceae sp.	0.000	0.117	0.000	0.114
			Pochonia sp.	0.000	0.148	0.000	0.041
			Aspergillus versicolor	0.000	0.187	0.000	0.000
			Microdochium phragmitis	0.000	0.158	0.017	0.000
			Glomerellaceae sp.	0.080	0.089	0.000	0.000
			Beauveria sp.	0.000	0.127	0.000	0.000
			Saccharomyces sp.	0.000	0.048	0.013	0.050
			Cenococcum geophilum	0.000	0.102	0.000	0.000
			Tetracladium sp.	0.000	0.063	0.019	0.000
			Alternaria sp.	0.000	0.064	0.011	0.000

		Acarosporaceae sp.	0.000	0.000	0.000	0.067
		Penicillium catenatum	0.000	0.058	0.000	0.000
		Caloplaca hanneshertelii	0.000	0.055	0.000	0.000
		Pleosporales sp.	0.000	0.054	0.000	0.000
		Sordaria sp.	0.000	0.049	0.000	0.000
		Lecanora sp.	0.000	0.000	0.047	0.000
		Verrucaria ditmarsica	0.042	0.000	0.000	0.000
		Mycosphaerellaceae sp.	0.035	0.000	0.000	0.000
		Diutina catenulata	0.000	0.030	0.000	0.000
		Phaeosphaeria triglochinicola	0.000	0.000	0.000	0.029
		Capnodiales sp.	0.000	0.025	0.000	0.000
		Helotiaceae sp.	0.000	0.000	0.016	0.000
		Austroplaca darbishirei	0.000	0.016	0.000	0.000
		Debaryomyces sp.	0.000	0.014	0.000	0.000
		Lecidella stigmatea	0.000	0.012	0.000	0.000
		Lasiodiplodia sp.	0.000	0.012	0.000	0.000
		Xylaria apiculata	0.000	0.012	0.000	0.000
		Lecanora dispersa	0.000	0.009	0.000	0.000
		Hanseniaspora sp.	0.000	0.008	0.000	0.000
	Basidiomycota	*Glaciozyma antarctica*	0.799	0.060	0.000	0.000
		Malassezia restricta	0.000	0.134	0.000	0.000
		Cutaneotrichosporon debeurmannianum	0.000	0.125	0.000	0.000
		Typhula micans	0.000	0.000	0.000	0.105
		Sterigmatomyces halophilus	0.090	0.000	0.000	0.000
		Peniophoraceae sp.	0.000	0.000	0.000	0.082
		Malassezia arunalokei	0.000	0.072	0.000	0.000
		Malassezia sympodialis	0.070	0.000	0.000	0.000
		Leucosporidium creatinivorum	0.000	0.066	0.000	0.000
		Malasseziaceae sp.	0.000	0.043	0.020	0.000
		Cystofilobasidium infirmominiatum	0.000	0.058	0.000	0.000
		Psathyrellaceae sp.	0.000	0.021	0.000	0.000

			Hypholoma frowardii	0.000	0.000	0.020	0.000
			Glaciozyma martinii	0.000	0.000	0.016	0.000
			Rhodotorula diobovata	0.000	0.015	0.000	0.000
		Mortierellomycota	*Mortierella* sp.	0.000	22.792	20.763	0.000
			Mortierella alpina	0.000	0.328	13.347	16.475
			Mortierella turficola	0.000	0.000	0.000	16.171
			Mortierella antarctica	0.000	0.660	2.979	0.453
			Mortierella parvispora	0.000	0.104	0.000	0.000
			Mortierella elongatula	0.000	0.038	0.012	0.000
			Mortierella gamsii	0.000	0.041	0.000	0.000
		Chytridiomycota	*Betamyces* sp.	1.778	0.156	1.615	7.057
			Lobulomycetales sp.	0.413	0.068	1.589	1.109
			Spizellomycetales sp. 1	0.000	0.000	0.000	2.311
			Rhizophydiales sp.	0.905	0.000	0.123	0.670
			Chytridiales sp.	0.022	0.000	0.158	0.000
			Lobulomycetes sp.	0.000	0.000	0.022	0.000
		Rozellomycota	*Rozellomycota* sp. 1	23.846	0.000	0.000	0.000
		Zoopagomycota	*Syncephalis* sp.	0.000	0.000	0.716	0.000
		Basidiobolomycota	*Basidiobolales* sp.	0.000	0.000	0.012	0.000
		Monoblepharomycota	*Sanchytriaceae* sp.	0.000	0.046	0.000	0.000
BLASTn	Fungi	*Chytridiomycota*	*Spizellomycetales* sp. 2	18.420	0.301	1.428	5.784
			Neocallimastigales sp.	0.000	0.000	0.010	0.000
		Basidiomycota	*Agaricales* sp.	0.019	0.019	0.000	0.000
			Basidiomycota sp.	0.285	0.070	0.023	0.000
		Ascomycota	*Sordariales* sp.	0.000	0.000	0.013	0.252
		Rozellomycota	*Rozellomycota* sp. 2	0.000	0.000	0.000	0.032
		Mucoromycota	*Mortierellales* sp.	0.000	0.000	0.175	0.000
		Unknown	Fungal sp. 1	21.563	29.792	11.791	16.329
			Fungal sp. 2	23.204	3.046	19.103	22.008
			Fungal sp. 3	0.000	0.000	0.000	0.076

ASV = fungal sequence variant. Green indicates dominant, blue intermediate and orange minor relative abundance (see Methods).

Table S2. Shared fungal amplicon sequence variants (ASVs) detected in soil samples collected in Clearwater Mesa, James Ross Island, Antarctica

Site 1	Site 2	Site 3	Site 4	Site 1|Site 2	Site 1|Site 3	Site 1|Site 4	Site 2|Site 3	Site 2|Site 4	Site 3|Site 4	Site 1|Site 2|Site 3	Site 1|Site 3|Site 4	Site 2|Site 3|Site 4	Site 1|Site 2|Site 3|Site 4
Diaporthaceae sp.	*Physciaceae* sp.	*Syncephalis* sp.	*Mortierella turficola*	*Glaciozyma antarctica*	*Ciliophora* sp.	*Rozellomycota* sp.	*Mortierella* sp.	*Lecanicillium* sp.	*Helotiales* sp.	*Basidiomycota* sp.	*Rhizophydiales* sp.	*Penicillium nalgiovense*	Fungal sp. 2
Sterigmatomyces halophilus	*Didymosphaeriaceae* sp.	*Mortierellales* sp.	*Typhula micans*	*Glomerellaceae* sp.	*Chytridiales* sp.		*Microdochium phragmitis*	*Pochonia* sp.	*Sordariales* sp.			*Mortierella antarctica*	Fungal sp. 1
Malassezia sympodialis	*Ramalinaceae* sp.	*Lecanora* sp.	*Peniophoraceae* sp.	*Agaricales* sp.			*Alternaria* sp.	*Didymellaceae* sp.				*Mortierella alpina*	*Spizellomycetales* sp.
Verrucaria ditmarsica	*Aspergillus versicolor*	*Lobulomycetes* sp.	Fungal sp. 3				*Tetracladium* sp.					*Thelebolus balaustiformis*	*Betamyces* sp.
Mycosphaerellaceae sp.	*Malassezia restricta*	*Hypholoma frowardii*	*Acarosporaceae* sp.				*Malasseziaceae* sp.					*Saccharomyces* sp.	*Neoascochyta paspali*
	Beauveria sp.	*Helotiaceae* sp.	*Phaeosphaeria triglochinicola*				*Mortierella elongatula*						*Pseudogymnoascus* sp.
	Cutaneotrichosporon debeurmannianum	*Glaciozyma martinii*											*Penicillium* sp.
	Mortierella parvispora	*Bacillariophyta* sp.											*Antarctomyces* sp.
	Cenococcum geophilum	*Basidiobolales* sp.											*Lobulomycetales* sp.
	Malassezia arunalokei	*Neocallimastigales* sp.											*Candida parapsilosis*
	Leucosporidium creatinivorum												*Cladosporium* sp.
	Penicillium catenatum												*Aspergillaceae* sp.
	Cystofilobasidium infirmominiatum												
	Caloplaca hanneshertelii												
	Pleosporales sp.												
	Sordaria sp.												
	Sanchytriaceae sp.												
	Mortierella gamsii												
	Diutina catenulata												
	Capnodiales sp.												
	Psathyrellaceae sp.												
	Austroplaca darbishirei												
	Rhodotorula diobovata												
	Debaryomyces sp.												
	Lecidella stigmatea												
	Lasiodiplodia sp.												
	Xylaria apiculata												
	Lecanora dispersa												

Table S3. Ecological profiles obtained from the FUNGuild database and specific citations at generic level of the fungi detected in soil samples obtained from Clearwater Mesa, James Ross Island, Antarctic Peninsula.

	Relative abundance (%) of soil fungal ASVs				Trophic mode	Guild*
**Genus**	**Site 1**	**Site 2**	**Site 3**	**Site 4**		
Alternaria	0.000	0.064	0.011	0.000	Pathogenic-Saprotrophic-Symbiotic	Animal Pathogen-Endophyte-Plant Pathogen-Wood Saprotrophic
Antarctomyces	0.579	4.828	0.415	0.214	Saprotrophic	Undefined Saprotrophic
Aspergillus	0.000	0.187	0.000	0.000	Pathogenic-Saprotrophic	Animal Pathogen-Undefined Saprotrophic
Austroplaca	0.000	0.016	0.000	0.000	Symbiotic	Lichenized
Beauveria	0.000	0.127	0.000	0.000	Pathogenic	Animal Pathogen
Betamyces	1.778	0.156	1.615	7.057	Saprotrophic	Freshwater and Soil Saprotrophic (Lepelletier et al. 2014)
Caloplaca	0.000	0.055	0.000	0.000	Symbiotic	Lichenized
Candida	0.320	0.387	0.133	0.073	Pathogenic	Animal Pathogen
Cenococcum	0.000	0.102	0.000	0.000	Symbiotic	Ectomycorrhizal
Ciliophora	2.334	0.000	0.007	0.000	Symbiotic	Endophyte (Sun and Guo 2007)
Cladosporium	0.265	0.174	0.020	0.097	Symbiotic	Endophyte
Cutaneotrichosporon	0.000	0.125	0.000	0.000	Saprotrophic	Undefined Saprotrophic (Li et al. 2020)
Cystofilobasidium	0.000	0.058	0.000	0.000	Pathogenic	Fungal Parasite
Debaryomyces	0.000	0.014	0.000	0.000	Saprotrophic	Undefined Saprotrophic
Diutina	0.000	0.030	0.000	0.000	Pathogenic	Animal Pathogen (Ming et al. 2019)
Glaciozyma	0.799	0.060	0.016	0.000	Saprotrophic	Undefined Saprotrophic (Sampaio 2011)
Hanseniaspora	0.000	0.008	0.000	0.000	Pathogenic-Saprotrophic	Animal Pathogen-Fruits (Fleet et al. 2002; Saubin et al. 2020)
Hypholoma	0.000	0.000	0.020	0.000	Saprotrophic	Undefined Saprotroph
Lasiodiplodia	0.000	0.012	0.000	0.000	Pathogenic	Plant Pathogen
Lecanicillium	0.000	0.369	0.000	0.471	Pathogenic	Animal Pathogen
Lecanora	0.000	0.009	0.047	0.000	Symbiotic	Lichenized
Lecidella	0.000	0.012	0.000	0.000	Symbiotic	Lichenized

Leucosporidium	0.000	0.066	0.000	0.000	Saprotrophic	Soil Saprotrophic-Undefined Saprotrophic
Malassezia	0.070	0.206	0.000	0.000	Pathogenic	Animal Pathogen
Microdochium	0.000	0.158	0.017	0.000	Pathogenic-Symbiotic	Endophyte-Plant Pathogen
Mortierella	0.000	23.963	37.101	33.099	Saprotrophic-Symbiotic	Endophyte-Litter Saprotrophic-Soil Saprotrophic-Undefined Saprotrophic
Neoascochyta	1.663	10.873	0.991	0.535	Pathogenic	Plant Pathogen (Golzar et al. 2019)
Penicillium	1.334	13.206	1.369	0.699	Saprotrophic	Dung Saprotrophic-Undefined Saprotrophic-Wood Saprotrophic
Phaeosphaeria	0.000	0.000	0.000	0.029	Saprotrophic	Undefined Saprotrophic
Pochonia	0.000	0.148	0.000	0.041	Pathogenic	Animal Pathogen
Pseudogymnoascus	1.529	6.627	22.650	0.252	Pathogenic-Saprotrophic-Symbiotic	Animal pathogen-Soil Saprotrophic
Rhodotorula	0.000	0.015	0.000	0.000	Pathogenic-Saprotrophic	Animal Endosymbiont-Animal Pathogen-Endophyte-Plant Pathogen-Undefined Saprotrophic
Saccharomyces	0.000	0.048	0.013	0.050	Saprotrophic	Undefined Saprotrophic
Sordaria	0.000	0.049	0.000	0.000	Saprotrophic	Dung Saprotrophic -Wood Saprotrophic
Sterigmatomyces	0.090	0.000	0.000	0.000	Saprotrophic	Saprotrophic and halotolerant (Al-Tohamy et al. 2020)
Syncephalis	0.000	0.000	0.716	0.000	Pathogenic	Fungal Parasite
Tetracladium	0.000	0.063	0.019	0.000	Saprotrophic	Undefined Saprotrophic
Thelebolus	0.000	0.136	0.064	5.843	Saprotrophic-Symbiotic	Dung Saprotrophic-Endophyte-Undefined Saprotrophic
Typhula	0.000	0.000	0.000	0.105	Pathogenic	Plant Pathogen
Verrucaria	0.042	0.000	0.000	0.000	Symbiotic	Lichenized (Pykälä et al. 2020)
Xylaria	0.000	0.012	0.000	0.000	Saprotrophic-Symbiotic	Endophyte-Undefined Saprotrophic -Wood Saprotrophic

ASV = amplicon sequence variant.

References of Supplementary Table S3

Al-Tohamy R, Kenawy ER, Sun J, Ali SS (2020) Performance of a newly isolated salt-tolerant yeast strain *Sterigmatomyces halophilus* SSA-1575 for azo dye decolorization and detoxification. Front Microbiol 11:1163

Fleet G, Prakitchaiwattana C, Beh A, Heard G (2022) The yeast ecology of wine grapes. In: Ciani M (Ed) Biodiversity and Biotechnology of Wine Yeasts. Kerala, Research Signpost, 1-17

Golzar, H., Thomas, G., Jayasena, K.W. et al. (2019) *Neoascochyta* species cause leaf scorch on wheat in Australia. Australasian Plant Dis Notes 14:1-5

Frédéric Lepelletierab Karpov SA, Alacid E et al. (2014) *Dinomyces arenysensis* gen. et sp. nov. (*Rhizophydiales*, *Dinomycetaceae* fam. nov.), a chytrid infecting marine dinoflagellates. Protist 165:230-244

Li AH, Yan FX, Groenewald M et al. (2020) Diversity and phylogeny of basidiomycetous yeasts from plant leaves and soil: Proposal of two new orders, three new families, eight new genera and one hundred and seven new species. Stud Mycol 96:17-140

Ming C, Huang J, Wang Y et al. (2019) Revision of the medically relevant species of the yeast genus *Diutina*. *Med Mycol* 57:226-233

Pykälä J, Kantelinen A, Myllys L (2020) Taxonomy of *Verrucaria* species characterized by large spores, perithecia leaving pits in the rock and a pale thin thallus in Finland. MycoKeys 72:43

Sampaio J P (2011). *Leucosporidium* Fell, Statzell, IL Hunter & Phaff (1969). In The Yeasts, 1485-1494, Elsevier

Saubin M, Devillers H, Proust L (2020) Investigation of genetic relationships between *Hanseniaspora* species found in grape musts revealed interspecific hybrids with dynamic genome structures. Front Microbiol 10:2960

Sun X, Guo LD (2007) Endophytic fungi VI. *Ciliophora quercus* sp. nov. from China. Nova Hedwigia 85:403-406

Table S4. Comparison of fungal sequence diversity present in soils and lake sediments on Clearwater Mesa, James Ross Island.

Type and local	Diversity indices					Citation
	Number of fungal ASVs	Number of DNA reads	Fisher's-α (diversity)	Margalef's (richness)	Simpson's (dominance)	
**Soils**						
Site 1	25	15,636	10.7	5.21	0.81	Current study
Site 2	59	56,473	60.43	12.6	0.83	
Site 3	39	38,575	23.51	8.25	0.84	
Site 4	31	17,090	15.39	6.51	0.86	
**Lake sediments**						
Lilia	84	32,428	247.7	18.02	0.77	Gonçalves et al. (2022)
Cecilia	64	27,730	76.66	13.68	0.81	
Soledad	58	28,571	57.66	12.38	0.87	
Adriana	43	22,012	28.61	9.12	0.68	
Katerina	51	54,939	22.1	9.44	0.79	de Souza et al. (2022)
Florencia	171	46,813	559.7	32.09	0.92	

ASV = amplicon sequence variant.
